# Editorial: Closed-loop iterations between neuroscience and artificial intelligence

**DOI:** 10.3389/fnsys.2022.1002095

**Published:** 2022-12-19

**Authors:** Jinyu Li, Alexey Zaikin, Xiaochun Zhang, Shangbin Chen

**Affiliations:** ^1^Britton Chance Center for Biomedical Photonics, Wuhan National Laboratory for Optoelectronics-Huazhong University of Science and Technology, Wuhan, China; ^2^Institute for Women's Health and Department of Mathematics, University College London, London, United Kingdom; ^3^Department of Neurotechnology, Lobachevsky State University of Nizhny Novgorod, Nizhny Novgorod, Russia; ^4^Centre for Analysis of Complex Systems, Sechenov First Moscow State Medical University, Moscow, Russia; ^5^Federal Research Center Institute of Applied Physics of the Russian Academy of Sciences, Nizhny Novgorod, Russia; ^6^Department of Radiology, Guangzhou Women and Children's Medical Center, Guangzhou, China

**Keywords:** neuroscience, artificial intelligence, brain-inspired intelligence, computational model, neural network

“There is no scientific study more vital to man than the study of his own brain. Our entire view of the universe depends on it.” No one will doubt the importance of neuroscience addressed by Francis Crick. Neuroscience, studying the structure and function of the nervous system, is the central focus of the 21^st^ century. The brain is the only known example of truly general intelligence, which is of great enlightening significance to the study of artificial intelligence (AI) (Potter, 2007; Hassabis et al., 2017). Many AI researchers continue to look to neuroscience as a source of inspiration and insight (Ullman, 2019). Also, AI is rapidly becoming a valuable tool for neuroscience, helping to analyze various neural data and understand how the brain computes (Landhuis, 2020; Richards et al., 2022). Thus, the two synergistic fields have been keeping deep communication and exchange (Savage, 2019; Ito et al., 2022).

“If the human brain were so simple that we could understand it, we would be so simple that we couldn't.” This interesting quote from Emerson M. Pugh reflects our current position in trying to understand the human brain. Indeed, it is unclear how our brain works. We require multi-disciplinary inputs and efforts to unravel its intrinsic complexity. The mutual reinforcement between neuroscience and AI should be a closed loop for linking mind and machine. Computational neuroscience (Wang et al., 2020) could bridge these two fast-developing fields through adequate models representing and simulating the brain's unique architecture and functions as shown in Figure 1. The biophysics and algorithms obtained in neuroscience are crucial to implement brain-inspired intelligence. In turn, the AI system based on artificial neural network (ANN) inspired by the nervous system is being applied back to parse and interpret the brain. The full closed-loop may be organized as iterations and thus benefit both neuroscience and AI.

**Figure 1:**
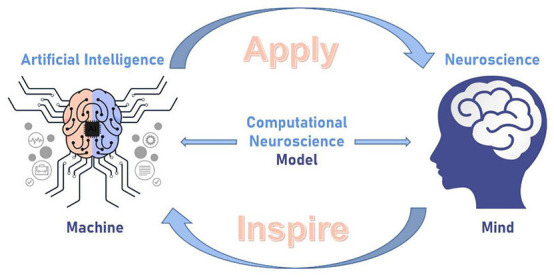
Schematic diagram of closed-loop iteration between neuroscience and artificial intelligence.

This Research Topic aims to provide a state-of-the-art review of computational neuroscience, including the development of AI inspired by neuroscience, the application of AI in brain research, and the closed-loop iterations between neuroscience and AI. So far, this Research Topic has collected 6 articles (5 original researches and 1 review) that represent the collaboration between AI and neuroscience.

There are 2 papers hitting on the topic of AI inspired by neuroscience. Zhao and Zeng proposed a brain-inspired synaptic pruning (BSP) algorithm, which can effectively modulate neural network architecture by pruning redundant synapses while retaining valid synapses during the learning process (Zhao and Zeng). Experimental results demonstrated that the BSP algorithm can significantly compress the network, showing its strengths and effectiveness in different sizes and the number of training samples on the network. Similarly, Liang and Zeng constructed a brain-inspired impulse neural network with the mechanism of human creativity, which can compose melodies with different styles of composers or genres (Liang and Zeng). It has been the first attempt to create musical melodies using biologically plausible ANN with the spike-timing-dependent plasticity learning rule. These two studies show that AI inspired by brain structures and functions is promising and moving toward human-like intelligence.

There are 4 papers focusing on the application of AI in neuroscience. (1) A detailed neocortical network model (also ANN) was presented to compare the capacities for signal processing between the human and rodent (Zhang et al.). The computational modeling suggests that the unique membrane properties in human neocortical neurons might improved the signal transmission accuracy. (2) Using electroencephalography (EEG) as input data, a machine learning method called random forest was used to detect schizophrenia (Vázquez et al.). The computational algorithm not only produces a diagnostic recommendation, but also helps the clinicians to locate key areas and/or connections in the brain associated with schizophrenia. (3) An ANN was pre-trained and exploited as a classifier achieving 74% accuracy on the EEG data of newly recruited subjects (Kuc et al.). This may greatly facilitate the development of brain-computer interfaces (BCI) by reducing the reliance of complex decoding algorithms. (4) The last review article comprehensively summarized the applications of reinforcement learning in BCI (Girdler et al.). Various reinforcement learning-based neural decoders have demonstrated impressive results and can be seamlessly integrated into more versatile and realistic BCIs. The aforementioned papers emphasize that the AI approaches are key techniques to make more discoveries and understand brain, even profit neural diseases and robotics.

Although there is no article presenting a complete closed-loop iteration in current Research Topic, this will potentially be a great work in the future. More recently, a single cortical neuron has been well approximated as a 5–8 layer deep ANN (Beniaguev et al., 2021). In addition, a general ANN has been optimized by introducing the principles of synaptic plasticity named group responsibility for adjusting the propagation of error signals (Dellaferrera et al., 2022). AI and improved microscopy have made it feasible to parse the connectome of the nervous system at ever-higher resolution (Landhuis, 2020). Just as Lichtman says, “Machines are learning to be smarter by studying the wiring of machines that are fundamentally smarter-biological machines.” No doubt, the closed-loop iterations between neuroscience and AI is a useful research paradigm for closing the gap between human intelligence and machine intelligence (Roy et al., 2019).

## Author contributions

All authors listed have made a substantial, direct, and intellectual contribution to the work and approved it for publication.
